# The Use of Online Consultation Systems or Remote Consulting in England Characterized Through the Primary Care Health Records of 53 Million People in the OpenSAFELY Platform: Retrospective Cohort Study

**DOI:** 10.2196/46485

**Published:** 2024-09-18

**Authors:** Martina Fonseca, Brian MacKenna, Amir Mehrkar, The OpenSAFELY Collaborative, Caroline E Walters, George Hickman, Jonathan Pearson, Louis Fisher, Peter Inglesby, Seb Bacon, Simon Davy, William Hulme, Ben Goldacre, Ofra Koffman, Minal Bakhai

**Affiliations:** 1 NHS England London United Kingdom; 2 Bennett Institute for Applied Data Science Nuffield Department of Primary Care Health Sciences University of Oxford Oxford United Kingdom; 3 See Acknowledgments Oxford United Kingdom

**Keywords:** online consultation system, remote monitoring, triage, primary care research, health informatics, general practice, digital primary care, electronic health record coding, OpenSAFELY, trusted research environment

## Abstract

**Background:**

The National Health Service (NHS) Long Term Plan, published in 2019, committed to ensuring that every patient in England has the right to digital-first primary care by 2023-2024. The COVID-19 pandemic and infection prevention and control measures accelerated work by the NHS to enable and stimulate the use of online consultation (OC) systems across all practices for improved access to primary care.

**Objective:**

We aimed to explore general practice coding activity associated with the use of OC systems in terms of trends, COVID-19 effect, variation, and quality.

**Methods:**

With the approval of NHS England, the OpenSAFELY platform was used to query and analyze the in situ electronic health records of suppliers The Phoenix Partnership (TPP) and Egton Medical Information Systems, covering >53 million patients in >6400 practices, mainly in 2019-2020. Systematized Medical Nomenclature for Medicine–Clinical Terminology (SNOMED-CT) codes relevant to OC systems and written OCs were identified including *eConsultation*. Events were described by volumes and population rates, practice coverage, and trends before and after the COVID-19 pandemic. Variation was characterized among practices, by sociodemographics, and by clinical history of long-term conditions.

**Results:**

Overall, 3,550,762 relevant coding events were found in practices using TPP, with the code *eConsultation* detected in 84.56% (2157/2551) of practices. Activity related to digital forms of interaction increased rapidly from March 2020, the onset of the pandemic; namely, in the second half of 2020, >9 monthly *eConsultation* coding events per 1000 registered population were registered compared to <1 a year prior. However, we found large variations among regions and practices: December 2020 saw the median practice have 0.9 coded instances per 1000 population compared to at least 36 for the highest decile of practices. On sociodemographics, the TPP cohort with OC instances, when compared (univariate analysis) to the cohort with general practitioner consultations, was more predominantly female (661,235/1,087,919, 60.78% vs 9,172,833/17,166,765, 53.43%), aged 18 to 40 years (349,162/1,080,589, 32.31% vs 4,295,711/17,000,942, 25.27%), White (730,389/1,087,919, 67.14% vs 10,887,858/17,166,765, 63.42%), and less deprived (167,889/1,068,887, 15.71% vs 3,376,403/16,867,074, 20.02%). Looking at the *eConsultation* code through multivariate analysis, it was more commonly recorded among patients with a history of asthma (adjusted odds ratio [aOR] 1.131, 95% CI 1.124-1.137), depression (aOR 1.144, 95% CI 1.138-1.151), or atrial fibrillation (aOR 1.119, 95% CI 1.099-1.139) when compared to other patients with general practitioner consultations, adjusted for long-term conditions, age, and gender.

**Conclusions:**

We successfully queried general practice coding activity relevant to the use of OC systems, showing increased adoption and key areas of variation during the pandemic at both sociodemographic and clinical levels. The work can be expanded to support monitoring of coding quality and underlying activity. This study suggests that large-scale impact evaluation studies can be implemented within the OpenSAFELY platform, namely looking at patient outcomes.

## Introduction

### Background

The National Health Service (NHS) England Digital First Primary Care (DFPC) program [1] has led the work on enabling the implementation and improvement of the use of digital tools in general practice, including the use of online consultation (OC) systems (see Textbox 1 for OC system clarification) and video consultation (VC) systems. The NHS Long Term Plan [2], published in January 2019 before the COVID-19 pandemic, committed to ensuring that every patient in England has the right to digital-first primary care by 2023-2024; the 5-year general practitioner (GP) contract reform framework to support the NHS Long Term Plan specifically committed for all practices to offer OC and VC systems by 2021. Legislation that came into force in October 2021 requires all NHS GP practices to make an OC system available to their patients [3]. The COVID-19 pandemic response significantly accelerated the adoption and need for such systems. An internal NHS England data collection of information provided by OC system suppliers (rapidly established in April 2020) [4] showed that as of June 21, 2021, approximately 79% of the practices in England had OC system capability in place. This was believed to stand closer to 95% when considering gaps in data collection [5]. In early 2021, there were approximately 10 weekly OC submissions per 1000 population, although underscored by local variation. Most recently, the NHS England Delivery Plan for Recovering Access to Primary Care, published in early May 2023, outlined the ambition for general practices to move to a fairer, safer, and more sustainable model of general practice termed the Modern General Practice Access Model [6]. An important enabler of this model is the use of OC systems for improving access and supporting care navigation and triage in general practice.

**Online consultation (OC) systems and electronic health record systems.**

**OC system**

An OC system is an online facility that allows a patient or carer to seek advice or information related to the patient’s health or to make a clinical or administrative request through completing an electronic form. The written information provided by patients or carers about the issue for which they are seeking help enables practices to prioritize patient care based on clinical need and to ensure that care is offered by the right member of staff or service and in the right way. The mode of response is based on the clinical need, circumstances, and patient’s communication preferences. While OC systems offer the patient or carer a way to access care, clinical staff decide (taking into account patients’ preferences) whether the patient will be responded to via a telephone call, invited to a video consultation, invited for a face-to-face appointment, or whether they will receive a written response in electronic form (eg, SMS text message or online message) [7]. A written OC is a 2-way written exchange between a health care professional and a patient using an online medium (such as a web platform or SMS text messaging). OC systems enable digital forms of patient-practice interaction and have only recently been more widely implemented.

**Electronic health record systems**

This study also mentions electronic health record systems. These are well established and are used as a much broader means to record and identify activity electronically, mainly through coding terminology. These contain results of clinical and administrative encounters between a provider (physician, nurse, and others) and a patient that occur during episodes of patient care (it is not specific to recording digital forms of patient-practice interaction, although that would be in scope [8]).

Given the rapid nationwide adoption and use of OC systems and other digital tools (including alongside and in response to the COVID-19 pandemic), there is a need to better understand how they are used, implemented, and generate impact. Monitoring and evaluation have been stood up by the DFPC team as well as independent studies. Specifically, commissioned work includes research questions comparing different models of implementation and different types of OC systems; analysis of patient experience; and analysis of the impact of digital tools on outcomes such as prescribing patterns, accident and emergency department attendances, and emergency admissions [9,10]. However, activity-monitoring data sets such as General Practice Appointments Data (GPAD) [11] or the internal NHS England data collection from OC system suppliers are aggregate and do not capture sociodemographics, clinical history, or patient pathways. The data available in GPAD are classified as experimental data due to variations in practice coverage. GPAD presents details of patient appointments that are recorded in the general practice appointments system, rather than the totality of interactions. As such, it cannot be considered to be a complete view of general practice activity. Specifically, it does not currently capture all triage activity or all appointments following from online requests to practices because they are often managed within other IT products. The GPAD data set also does not currently include all enhanced-access evening and weekend appointments, which are managed by other appointment books. In terms of consultation appointment mode, it only includes a broad *video or online* category (including VCs, videoconferencing, written OCs, and the like), which is returned with variable completion and standardization. By contrast, more targeted pilots and evaluation studies have been designed that do access richer quantitative or qualitative information [9,10,12,13], but these tend to be localized to a single supplier or set of general practices; therefore, they may not be nationally representative or provide a full picture along the pathway.

### Objectives

OpenSAFELY is a new secure analytics platform for electronic health records (EHRs) in the NHS, created to deliver urgent insights during the global COVID-19 emergency. The platform uses a novel approach for enhanced security and timely data access that avoids the need to migrate large volumes of disclosive pseudonymized patient data outside of the secure environments managed by the EHR software companies (eg, The Phoenix Partnership [TPP] and Egton Medical Information Systems [EMIS]); instead, it relies on trusted analysts to run computations and analysis on near–real-time pseudonymized patient records still held inside the data centers and secure cloud environments of EHR companies. With the approval of NHS England, we conducted a service evaluation using the NHS England OpenSAFELY COVID-19 research platform. In this particular study, we explore EHR coding activity that is related to the use of OC systems and written OCs (that is, responses delivered by SMS text messages or online messages) in general practices via OpenSAFELY. By using primary care EHR system data (Textbox 1), it is possible to leverage nationally representative, longitudinal patient cohort data regarding clinical and administrative encounters and to analyze this in the context of other factors such as geography, sociodemographics, clinical characteristics, and other health care interactions. Given the limited pre-existing standardization and insight into the coding of submissions received via an OC system and the coding of the mode of consultation, we set out to focus on the following aims:

- Understanding OC system and written OC coding use and prevalence in primary care records by codes of interest (OC system-relevant as shorthand)
- Understanding the variation in OC system–relevant coding use over time, before and after the start of the COVID-19 pandemic, and in terms of interpractice variation
- Understanding broad demographics and past clinical history of patients with OC system–relevant coding activity

This exploratory analysis may help inform further research and evaluation questions and their feasibility, including large-scale impact evaluation studies.

## Methods

### Study Design

We conducted a retrospective cohort study using general practice primary care EHR data from all GP practices in England with EHR vendors TPP and EMIS as suppliers. This analysis project is part of a “ways of working” pilot to onboard into OpenSAFELY any new approved users or researchers (including NHS England analysts) [14].

### Data Source

All data were linked, stored, and analyzed securely within the OpenSAFELY platform [15], which is a data analytics platform created with the approval of NHS England to address urgent COVID-19 research questions. Data records used in this study are pseudonymized general practice primary care EHR data from practices in England that are supplied by the vendors TPP and EMIS. These contain data such as diagnoses, medications, and sociodemographic characteristics. Similarly pseudonymized data sets from other data providers are securely provided to the EHR vendor and linked to the primary care data, such as information on care home status. No free-text data are included.

The TPP database analyzed via OpenSAFELY (OpenSAFELY-TPP) is based on 24.2 million people currently registered with 2546 GP surgeries using TPP SystmOne software, while the EMIS database analyzed within OpenSAFELY (OpenSAFELY-EMIS) is based on 32.6 million people currently registered with 3821 GP surgeries using EMIS. Together, these represent approximately 99% of the practices. Most of the outcomes in this study explore OpenSAFELY-TPP, although a more fixed-scope overall coding use and prevalence characterization was extended to OpenSAFELY-EMIS as well, based on what was feasible in its early operational days (Multimedia Appendix 1).

OpenSAFELY-TPP and OpenSAFELY-EMIS were used side by side to identify the use of OC-relevant activity in the period surrounding the pandemic start (from January 2019 to December 2020) in terms of individual code use and practice coverage. OpenSAFELY-TPP, covering approximately 40% of practices, was used to understand trends in coding activity over time (before and after the start of the COVID-19 pandemic), interpractice variation, associations with sociodemographic factors, and associations with clinical history factors.

For benchmarking and triangulation, data from the national OC and VC system supplier collection are also used [4]. This data collection was stood up rapidly at the start of the pandemic and includes aggregate use data taken directly from the participating OC system suppliers. This contains daily information from August 2020 onward, derived from the daily collection files. It can be extended as a weekly trend back to April 2020. No information on demographics, clinical history, or pathway is part of its specifications. On completeness, an audit undertaken on March 31, 2021, suggested that approximately an additional 10% of practices were using an OC system supplier that did not contribute to the national collection. As of September 2021, there were 5 out of 20 suppliers that had not submitted their data, meaning that metrics on total national use will be understated, which should be taken into account when benchmarking. However, the situation had been evolving because several suppliers were working toward submitting their data. Practices using either TPP or EMIS as their EHR system were identified by linking to a separate data collection, Patient Online Management Information (POMI) [16].

### Coding Systems

In general practice, staff record information about patients using clinical coding systems such as Systematized Medical Nomenclature for Medicine–Clinical Terminology (SNOMED CT) and the Dictionary of Medicines and Devices. The TPP system is fully compliant with SNOMED-CT, with GPs using it in their front-end interactions with EHR systems, having previously used Clinical Terms Version 3 (CTV3) before the NHS-wide standard was adopted. OpenSAFELY can query the records using either CTV3 or SNOMED-CT, which allows flexibility on querying some past activity that cannot be easily mapped to SNOMED-CT.

### Approach to Deciding Codes for Interrogation

We could not ascertain the existence of a nationally consistent and standardized codelist for activity associated with OC systems or with the carrying out of a written OC, although NHS England is undertaking work to facilitate standardized coding; for example, SNOMED-CT codes [17] for OC systems were agreed upon in 2022. The historic absence of such codelists relates to a number of reasons, including the recency and pace of technology implementation and adoption, the lack of standardization of terminology and appropriate codes around digital forms of interaction with a practice (by route and mode), and the disparity between supplier systems and templates. As such, a custom SNOMED-CT codelist was created on OpenCodelists with existing relevant codes. This is available for inspection and reuse by anyone [18]. The codes are also provided in Table 1.

In terms of criteria, the term *online consultations* is ambiguous and can include submissions and requests received from patients via an OC system (route of access) or a consultation mode using written electronic messaging (appointment mode). Codes of interest were those deemed to be associated with either submissions made using an OC system (route) or written OCs (mode). Keywords included in the search were “consultation (procedure),” “econsultation,” “indirect encounter,” “online,” “remote triage,” “telemedicine,” and “telepractice.”

**Table 1:** The short-listed read codes in Systematized Medical Nomenclature for Medicine–Clinical Terminology (SNOMED-CT; the codelist builder list is available [18]).

SNOMED-CT code	Name (term)	CTV3^a^ or local TPP equivalent	TPP in active use in at least 1 practice
*1068881000000101*	eConsultation via web-based application (procedure)	Y1f3b	Yes
978871000000104	Consultation via multimedia (procedure)	—^b^	No
*448337001*	Telemedicine consultation with patient (procedure)	XaXcK	Yes
868184008	Telemedicine consultation with provider (procedure)	—	No
719407002	Remote nonverbal consultation (procedure)	—	No
763184009	Telepractice consultation (procedure)	—	No
*185320006*	Encounter by computer link (procedure)	.9N34 and 9N34.	Yes
1090371000000106	Referral to remote triage and advice service (procedure)	—	No
325951000000102	Remote assessment encounter type (record artifact)	—	No
*325871000000103*	Remote consultation encounter type (record artifact)	Y22b4	Yes
384131000000101	Remote encounter type (record artifact)	—	No
325911000000101	Consultation via multimedia encounter type	—	No
*699249000*	Alert received from telehealth monitoring system	XUman, XaX2B, 9G6..	Yes
*401271004*	Email sent to patient	XaIvi	Yes
325901000000103	Remote nonverbal consultation encounter type	—	No
325981000000108	Remote nonverbal assessment encounter type	—	No
325991000000105	Assessment via multimedia encounter type	—	No
854891000000104	Telehealth encounter type	—	No

^a^CTV3: Clinical Terms Version 3.

^b^Not applicable.

The codelist was developed using the following steps: (1) browse the SNOMED-CT Term Browser [9] for relevant keywords and *children* concepts (SNOMED-CT is a hierarchical terminology system where individual concepts can have *parent* concepts as well as *children* concepts that are essentially subtypes) and find their CTV3 equivalents (*refset*), if listed; (2) browse the NHS Digital CTV3 to SNOMED-CT Mapping Lookup [10] for relevant keywords and *children* concepts, and find their SNOMED-CT equivalents, if listed; (3) browse local TPP codes [11]; (4) pragmatically browse the literature, online resources, and white publications for further code indications [11-15]; and (5) obtain clinical and program input via the DFPC program on the initially found codes of interest (long list), as well as further codes, to arrive at a refined list.

### Recorded Code Activity and Practice Coverage Over Time (TPP)

The coding activity over time was characterized by individual code. The interpractice variation was also assessed.

#### Cohort

Using OpenSAFELY-TPP, for each week or month (period) of the study, the population of interest was defined as those aged ≥1 year (an early design choice to discard the possibility of 0 representing a null return), alive, and registered at the start of this period. Patients were assigned to the practice with which they were registered within this period. In turn, any activity (OC-relevant codes and GP consultations) a patient had in this period was assigned to their practice of registration. This yielded a study population of >23 million patients, relating to 2551 TPP practices.

#### Analysis

We extracted the number of times each code was recorded (1) during the period from January 2019 to December 2020 at monthly intervals and (2) from the period of January 6, 2020, to March 22, 2021, at weekly intervals. The OpenSAFELY weekly data were specifically generated for contextualization and benchmarking with a relevant weekly measure of OC submissions from a separate data source: the national OC or VC supplier data collection. The OpenSAFELY monthly data were used for all further analysis. Absolute count instances and rates per 1000 registered practice patient population were computed [19]. Practice coverage—the number of practices with at least 1 instance of the code over the 2-year period—was also calculated, at both national and regional levels [20]. We also calculated the rate at which certain codes were recorded per 1000 registered patients at a general practice level (among practices with any instance over the 2-year period) following the methods described in the NHS England National General Practice Improvement Programme [1]. We computed the deciles, median, and interdecile range for February, April, September, and December 2020 (ie, quarterly and for the start of the pandemic period).

The general practice consultation activity over this same period was also recorded for context; this uses a purpose-built function on OpenSAFELY rather than relying solely on counting code instances [19]: cohortextractor.patients.with_gp_consultations() captures GP-patient interactions, whether in person or by telephone or video call. The concept of a “consultation” in EHR systems is generally broader and might include things such as updating a telephone number with the receptionist. It captures events such as interactions and “consultations” differently from what is captured in the GPAD data set from GP appointment systems. It might also—but will not necessarily—capture interactions from OC-related events. In the analysis in this paper, the metrics and shorthand terminologies “GP consultation” and “GP-patient interaction”—whether in the Methods section, figures, tables, or descriptions—will refer to those obtained via the aforementioned OpenSAFELY EHR method, with the relevant caveats.

### Sociodemographics of Patients With Relevant Coding Activity (TPP)

Sociodemographic characteristics were characterized for patients with any OC-relevant coding instance.

#### Cohort

The population cohort was defined as all those registered with a single TPP GP practice between January 2019 and December 2020, resulting in a cohort of >20 million patients. The following characteristics were recorded, typically based on January 2019 status: ethnicity (based on ethnicity codelists), gender, age, care home status [21], household size, practice registered with and associated region, rurality of place of residence, disability status (learning disabilities and intellectual disabilities codelists created from the Quality and Outcomes Framework register), and Index of Multiple Deprivation 2019 quintile [22]. These characteristics come mainly from the primary care records themselves. While these are detailed and longitudinal, they can be incomplete regarding patient characteristics. Instead of excluding incomplete records, missing characteristic information is recorded as “unknown.”

The Index of Multiple Deprivation is a national statistic and the official measure of relative deprivation experienced by people living across England [22]. It is calculated for every lower layer super output area (approximately 1500 people) in England, and the overall rank is derived by combining and weighting the following domains: income; employment; health deprivation and disability; education, skills, and training; crime; barriers to housing and services; and living environment. In this study, each patient was assigned a deprivation rank and quintile according to the lower layer super output area they reside in.

#### Analysis

When producing summary statistics, the study population was divided into whether the patients had had any recorded OC-relevant coding instance (at least 1 match for any of the short-listed OC codes in the January 2019-December 2020 period). Summary statistics were also computed for patients who had had any GP consultation in this same period. For a given sociodemographic or geographic dimension at a time, we computed the following for the 2-year period: (1) the instance rate of codes (number of code instances, standardized per 1000 registered practice patient population), and (2) the coverage rate of codes (portion of population with at least 1 code instance).

### Clinical History of Patients With eConsultation Activity (TPP)

In further follow-up exploratory analysis, the clinical history of patients with eConsultation activity was investigated.

#### Cohort

The cohort included TPP practice–registered patients with a single practice between March 1, 2019, and February 28, 2021. Occurrences of the eConsultation code or of GP-patient interaction were recorded. Only patients in practices with non-nil eConsultation coverage were considered. Age and gender were captured. Clinical history flags per patient were dictated by whether each patient had any recorded occurrence from the individual codelists before March 2019. These were chosen to broadly align with the most prevalent long-term conditions according to the NHS England Population and Person Insight dashboard and framework [23]. The codelists were as follows: hypertension, asthma, chronic respiratory disease other than asthma, osteoarthritis, depression, diabetes, chronic heart disease, cancer, atrial fibrillation, stroke, peripheral arterial disease, heart failure, chronic kidney disease, and serious mental illness.

#### Analysis

Results, in terms of the historic prevalence of individual clinical conditions among those with an eConsultation code in either the *prepandemic* (March 2019-February 2020) or *pandemic* (March 2020-February 2021) period were captured. Prevalence figures were also tabulated alongside those of two relevant comparator subcohorts (only within practices using the eConsultation code at all in this period): (1) all the remaining practice population, and (2) the remaining practice population with a recorded activity of a GP consultation in this period. A multivariate logistic regression model was used to assess which clinical conditions were associated with higher adjusted odds of having had an eConsultation code recorded among the population in those practices that had had a GP consultation or eConsultation coding activity. Age groups and gender were also included as a first order case-mix adjustment. Further confounding (whether from interactions or other omitted characteristics) may remain.

### Recorded Code Activity and Practice Coverage (EMIS and TPP)

For the analysis of coding activity leveraging both EMIS and TPP (about 99% of the GP practices), a more fixed-scope exploration was implemented as a compromise, given the OpenSAFELY-EMIS functionality and server availability, which was in its earlier stages. The results can be found in Multimedia Appendix 1 (“Coding activity prevalence [EMIS]”).

#### Cohort

We defined the population of interest as those aged ≥1 year; alive; and registered as of January 1, 2019. This resulted in cohorts of >23 million and >30 million patients for TPP and EMIS, respectively. Patients were assigned to the practice they were registered with at the start. In turn, any activity (codes associated with OC systems, remote consultations, or with the EHR query on GP consultations) a patient had in this 2-year period was assigned to the initial practice of registration, rather than having activity reassigned to a new practice if the patient moved (this was necessary due to practical OpenSAFELY-EMIS functionality considerations). This differs from the TPP coverage analysis in the previous subsections, which reflected month-on-month fluctuations in patient registration for each patient and reassigned activity accordingly.

#### Analysis

A table comparing EMIS and TPP statistics under similar conditions is presented in Multimedia Appendix 1. We show the number of times each code was recorded during the period from January 2019 to December 2020, aggregated over the 2 years. Values are given per 1000 registered population and also per 1000 registered patient population in practices where the code was recorded. To mitigate practice disclosure, results are not shown where a code was found in <5 practices.

### Software and Reproducibility

Data management was performed using Python (version 3.8.0; Python Software Foundation), with analysis carried out using R (R Foundation for Statistical Computing). All code for the OpenSAFELY platform for data management, analysis, and secure code execution is shared for review and reuse under open licenses [24]. All project code used for data management and analysis is also shared openly for review and reuse under the Massachusetts Institute of Technology license [25]. The developed codelist is publicly available as well [18]. The project code and codelists were transmitted to the OpenSAFELY-TPP platform within the TPP secure environment and to the OpenSAFELY-EMIS platform within the EMIS secure environment for local execution against pseudonymized data, that is, without the need for the researcher to locally see or move pseudonymized records. After a review for the disclosiveness of aggregate results in a secure server, the cleared aggregate results were released into final openly available outputs in the project repository. Although detailed pseudonymized patient data are potentially reidentifiable and therefore not shared (nor was it directly viewed when undertaking this research, given the platform design outlined previously), further queries to, or analysis against, the same pseudonymized records can be made through a new project request to the OpenSAFELY framework [14].

### Ethical Considerations

NHS England is the data controller for OpenSAFELY-EMIS and OpenSAFELY-TPP, EMIS and TPP are the data processors, and all study authors using OpenSAFELY have the approval of NHS England. This implementation of OpenSAFELY is hosted within EMIS and TPP environments that are accredited to the ISO 27001 information security standard and are NHS Information Governance Toolkit compliant [26,27].

Patient data have been pseudonymized for analysis and linkage using industry-standard cryptographic hashing techniques; all pseudonymized data sets transmitted for linkage onto OpenSAFELY are encrypted; access to the platform is via a virtual private network connection, restricted to a small group of researchers; the researchers hold contracts with NHS England and only access the platform to initiate database queries and statistical models; all database activity is logged; and only aggregate statistical outputs leave the platform environment following best practice for the anonymization of results, such as statistical disclosure control for low cell counts [28].

The OpenSAFELY research platform adheres to the obligations of the UK General Data Protection Regulation and the Data Protection Act 2018. In March 2020, the Secretary of State for Health and Social Care used powers under the UK Health Service (Control of Patient Information) Regulations 2002 to require organizations to process confidential patient information for the purposes of protecting public health, providing health care services to the public, and monitoring and managing the COVID-19 outbreak and incidents of exposure; this sets aside the requirement for patient consent [29].

Taken together, these provide the legal bases to link patient data sets on the OpenSAFELY platform. GP practices, from which the primary care data are obtained, are required to share relevant health information to support the public health response to the pandemic and have been informed of the OpenSAFELY analytics platform. This study has been approved by internal NHS England processes as per openly available policies [30,31]. NHS England service evaluations or audits in OpenSAFELY are currently not required to have ethics approval. This study was supported by MB, DFPC National Clinical Director, as senior sponsor.

### Patient and Public Involvement

We have developed a publicly available website [15] through which we invite any patient or member of the public to contact us regarding this study or the broader OpenSAFELY project.

## Results

The results are presented in the following subsections for TPP-based practices and cohorts. The results for the combined OpenSAFELY-TPP and OpenSAFELY-EMIS analysis can be found in Multimedia Appendix 1; an aid to interpretation is given in Textbox 2.

**Considerations when interrogating codes and coding activity.**

**Interpreting output charts and tables**

When interpreting output charts and tables, it is important to consider the following:

- All occurrences of codes are included, and they do not necessarily indicate unique or new events (eg, 1 patient encounter could generate several similar codes, 1 patient might have similar diagnoses recorded multiple times over time, or practices might import information in bulk).
- There might be other similar codes occurring in the data that are not included in the charts.
- Conversely, some codes are not exclusively used for the activity under study (eg, remote consultations can include a broader range of activity, such as telephone or video consultations).
- Not all codes represent activity occurring in general practice and may have been passed into the patient record from other services, including third-party systems.
- Some apparent changes may represent changes in coding behavior or displaced activities.
- Coding is dependent on manual input and therefore prone to inconsistency and gaps.

**Coding related to online consultation systems and the interpretation of recorded activity**

Coding related to online consultation (OC) systems and the interpretation of recorded activity is not straightforward. This is due to the following reasons:

- The use of OC systems and their national rollout across practices are fairly recent. These codes do not differentiate between requests made using an OC system (route of access) and written OC appointments (mode of consultation with a patient or carer). These codes do not differentiate between administrative and clinical activity.
- There are not yet specific Systematized Medical Nomenclature for Medicine–Clinical Terminology (SNOMED-CT) codes for OC submissions and written OC appointments, although these are currently in development. In this analysis, coding (and the clinical coding system) depends on the practice user, functionality, and user interface within the general practice IT clinical system, specific supplier technology, and its template implementation.
- The implementation of OC systems can differ among practices, both in terms of the patient journey (service model) and the underlying technology. Some practices manage online requests and subsequent appointments within their OC system rather than the GP IT clinical system. Therefore, the recording and nature of the (series of) codes generated will differ.
- The mode of making contact does not determine the mode of consultation; practices may only code the mode of consultation (appointment; eg, a telephone, video, or face-to-face appointment) rather than the route of contact. Where new codes have been created recently that are relevant to remote consultation or the use of OC systems, these are typically SNOMED-CT codes and will tend to not have a CTV3 equivalent unless a local code is defined. Nevertheless, TPP is still quite reliant historically on CTV3; therefore, the richness of recording will largely be in the legacy system (if the practice is using Systematized Medical Nomenclature for Medicine–Clinical Terminology, it may only do so via mapping to CTV3).
- In some cases, guidance is given for new forms of consultations to be recorded in annotated free-text fields of higher-level codes. Free-text–querying functionality is not currently available in OpenSAFELY.

### Weekly Coding Activity and Contextualization With Rapid Supplier Data Collection

**Figure 1:**
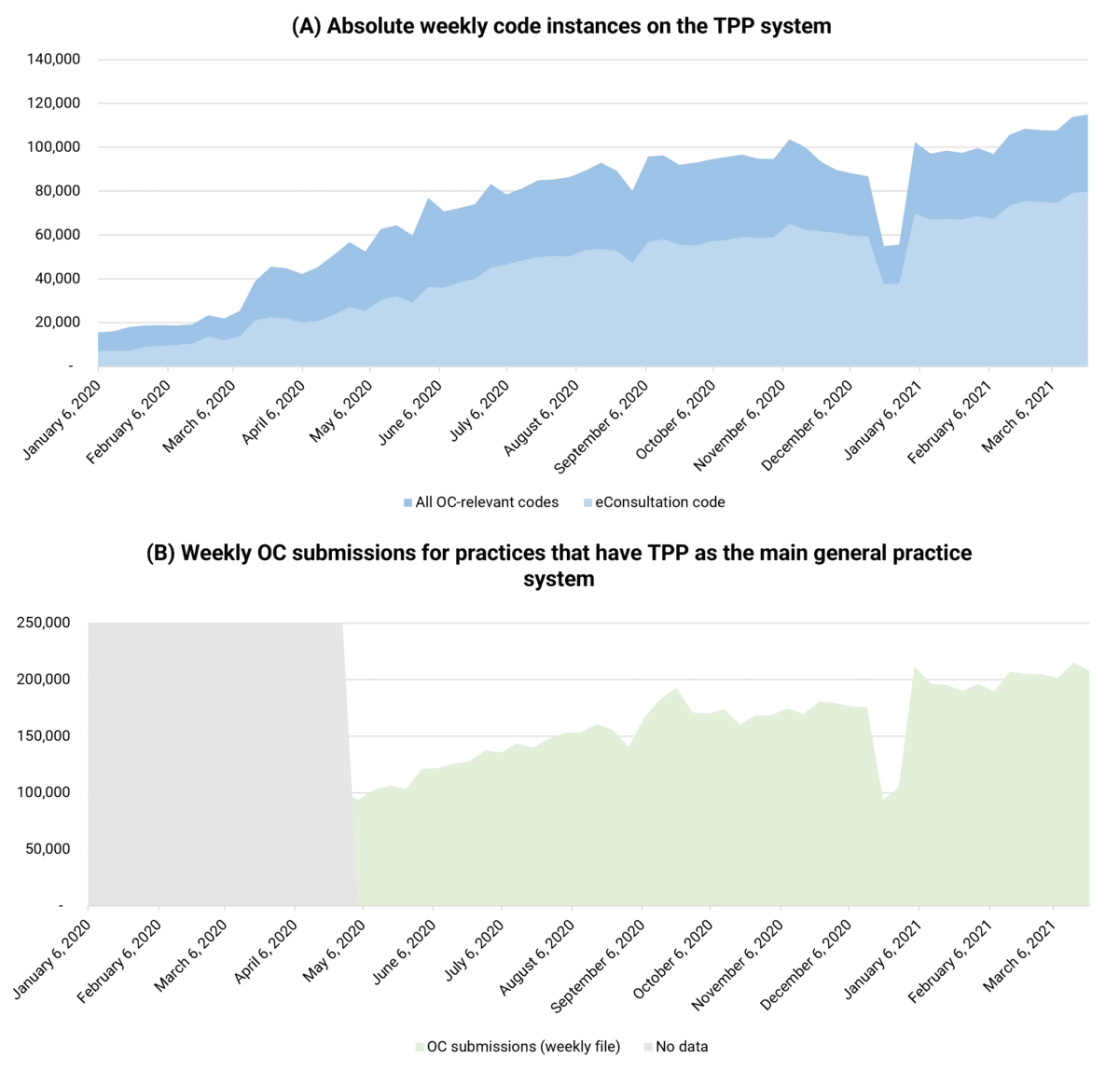
(A) Absolute weekly online consultation (OC) code instances in The Phoenix Partnership (TPP) system (source: OpenSAFELY-TPP). (B) Weekly OC submissions for practices that have TPP as the main general practitioner system (source: national rapid collection). Between 66 and 132 practices each week had no clear system associated in the patient web-based management information data collection and were not included.

Figure 1A depicts the absolute instances of the code eConsultation, as well as of all SNOMED CT codes combined. It reflects coding activity through all TPP practices during the period from January 6, 2020, to March 22, 2021. However, different practices may approach the coding of different activity associated with the use of OC systems differently, especially based on the OC system supplier in place, chosen OC pathways, and implementation maturity [32].

Separately, Figure 1B depicts the total OC submissions during the period from April 27, 2020, to March 22, 2021, according to the NHS England OC or VC data collection from system suppliers (rapid and aggregate) [4]. This is likely still an underestimation due to data completeness issues.

The shapes of the 2 graphs over time look the same in terms of peaks and troughs, and both have a similar overall significant rising profile. If we assume that each OC submission should generate at least 1 activity code in the primary care systems, then the data suggest that coding activity in general practice IT clinical systems is not fully tracking all OC activity. The 2 data sources do track different but related activity. This is addressed in the Methods and Discussion sections.

### Codes in Use and Practice Coverage

**Figure 2:**
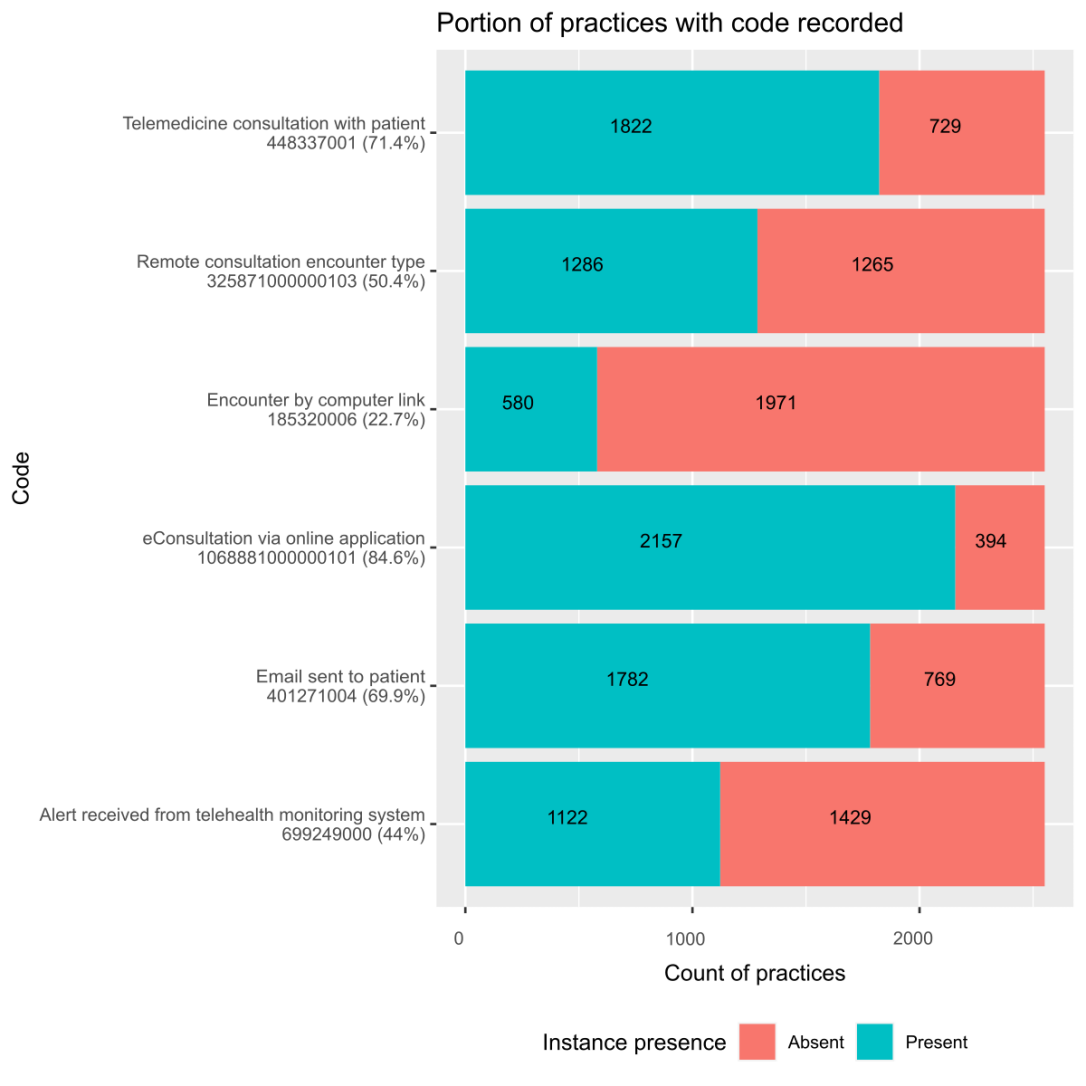
Portion of The Phoenix Partnership (TPP) practices with any recorded activity for online consultation–relevant codes (January 2019-December 2020). Codes with no activity at all have been omitted.

Figure 2 shows the portion of practices that had at least 1 instance of the respective SNOMED CT codes over the 2-year period. Breakdowns by region are presented in Figures S1 and S2 in Multimedia Appendix 1.

Of the 18 codes, 12 (67%) returned no results in TPP. The last column of Table 1 indicates this. The SNOMED CT codes for which instances *were* found also correspond to those where both (1) a CTV3 mapping was available when specifying the codelists, and (2) CTV3 querying had activity recorded (not shown). The practice coverage, in decreasing order, was as follows: *eConsultation via online application* (1068881000000101; 2157/2551, 84.56% of the practices), *telemedicine consultation with patient* (448337001; 1822/2551, 71.42%), *email sent to patient* (401271004; 1782/2551, 69.85%), *remote consultation encounter type* (325871000000103; 1286/2551, 50.41%), *alert received from telehealth monitoring system* (699249000; 1122/2551, 43.98%), and *encounter by computer link* (185320006; 580/2551, 22.74%).

### Monthly Trends in Coding Activity

**Figure 3:**
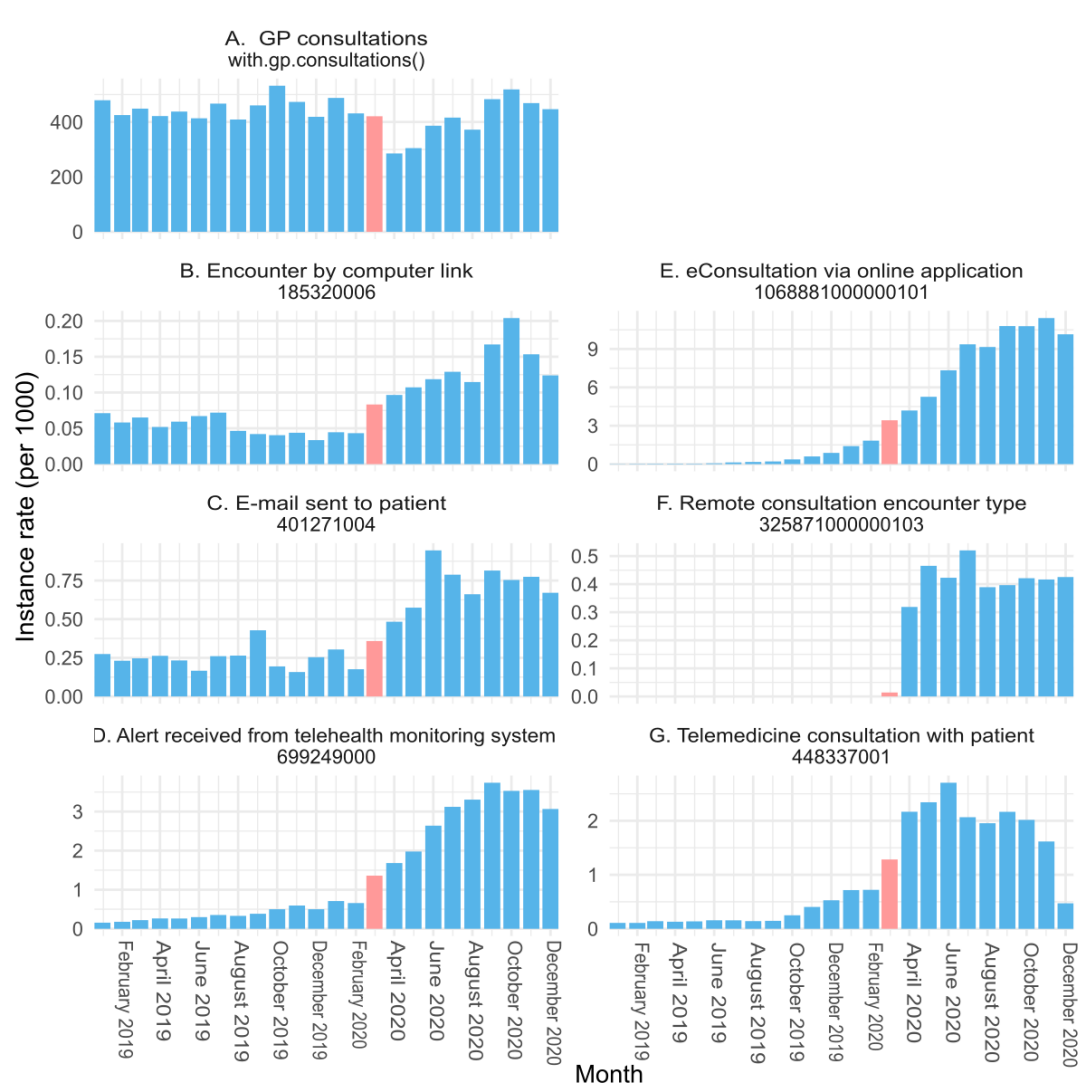
Monthly code instance rates per 1000 registered practice patient population of Systematized Medical Nomenclature for Medicine–Clinical Terminology (SNOMED CT) codes in The Phoenix Partnership (TPP) general practice (January 2019-December 2020). March 2020 data are indicated in pink. (A) General practitioner consultations, with.gp.consultations(). (B) Encounter by computer link, 185320006. (C) Email sent to patient, 401271004. (D) Alert received from telehealth monitoring system, 699249000. (E) eConsultation via web-based application, 1068881000000101. (F) Remote consultation encounter type, 325871000000103. (G) Telemedicine consultation with patient, 448337001.

While the previous figures focused on coverage, Figure 3 shows the monthly use of the various codes (coding activity) during the period from January 2019 to December 2020. Values are given as a rate (per 1000 cohort population). The entire cohort population is considered, rather than just patients in practices where each code was recorded. The rate of GP consultation events is also given for context (the practice coverage is near complete at >99%, as expected). Absolute counts are shown in Figure S3 in Multimedia Appendix 1.

The codes with the highest activity, ordered by highest monthly peak, are presented in Textbox 3. We have also plotted the monthly rate of (overall) GP consultations regardless of the modality in the TPP practices. This stood broadly at >400 consultations per 1000 patients in 2019. The dip is seen around April 2020. Recovery occurred, with October 2020 registering the second highest monthly rate (519 consultations per 1000 patients) after October 2019 (532 consultations per 1000 patients; Figure 3A).

**Codes with the highest activity, ordered by highest monthly peak.**

**Coding activity**

- The coding activity is shown in Figure 3 for: B) encounter by computer link C) email sent to patient; D) email sent to patient; E) eConsultation via online application; F) remote consultation encounter type; G) telemedicine consultation with patient.
- In order of highest to lowest activity:
  eConsultation via online application (1068881000000101): there was a peak of >10 monthly coding events per 1000 registered population in November 2020. This has increased rapidly from virtually none in early 2019.
  Alert received from telehealth monitoring system (699249000): there was a peak of >3.5 events per 1000 registered population in September 2020. This has increased rapidly compared to 2019. A first step change is seen around the start of the pandemic (February-March 2020.
  Telemedicine consultation with patient (448337001): there was a peak of >2.5 events per 1000 registered population in June 2020. Step changes from February to March and March to April 2020 are noticeable.
  Email sent to patient (401271004): there was a peak of close to 1 event per 1000 registered population in June 2020. Step changes from February to March and March to April 2020 are noticeable.
  Remote consultation encounter type (325871000000103): there was a peak of >0.5 events per 1000 registered population in July 2020. This is likely a new code; its use seems to have been first recorded in March 2020. This may relate to TPP introducing a local TPP [33] dedicated code that maps to this Systematized Medical Nomenclature for Medicine–Clinical Terminology code (Y22b4).
  Encounter by computer link (185320006): there was a peak of >0.2 events per 1000 registered population in October 2020. Its use seemed to be in slight decline in 2019 and then got a step increase from March 2020.

### Interpractice Variation in Monthly Coding Activity Trends

**Figure 4:**
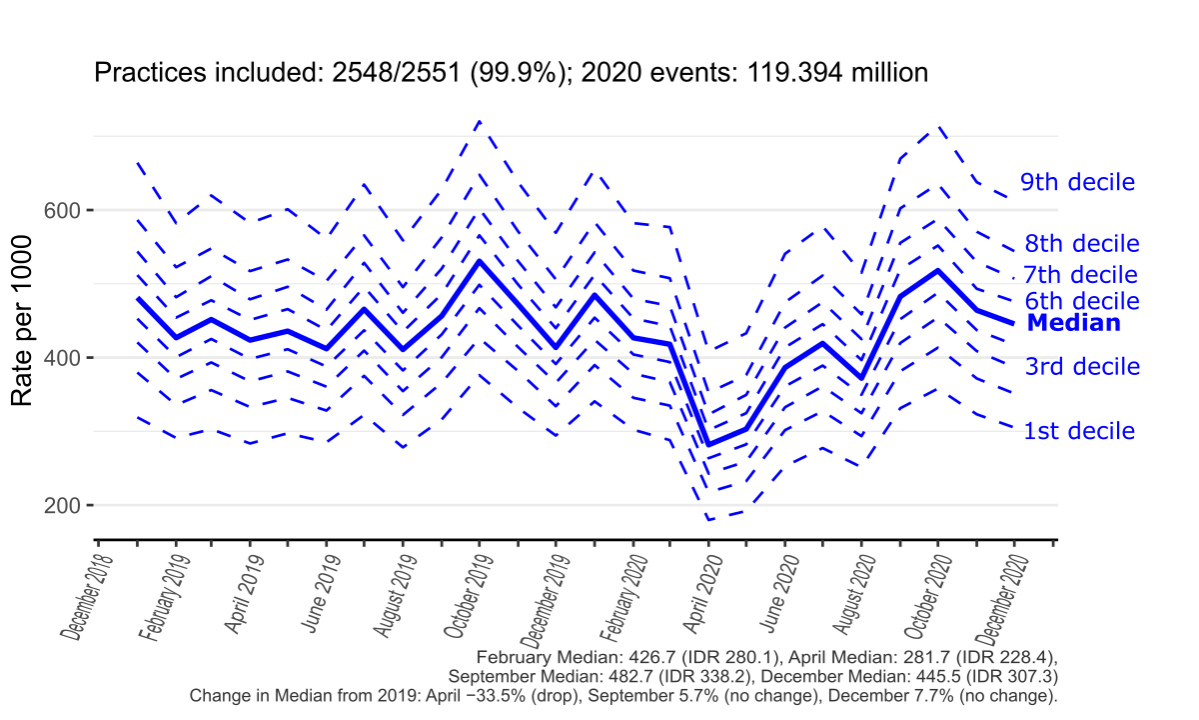
Contextual information. Recording of results from general practice consultations (any modality) in general practice (January 2019-December 2020). IDR: interdecile range.

**Figure 5:**
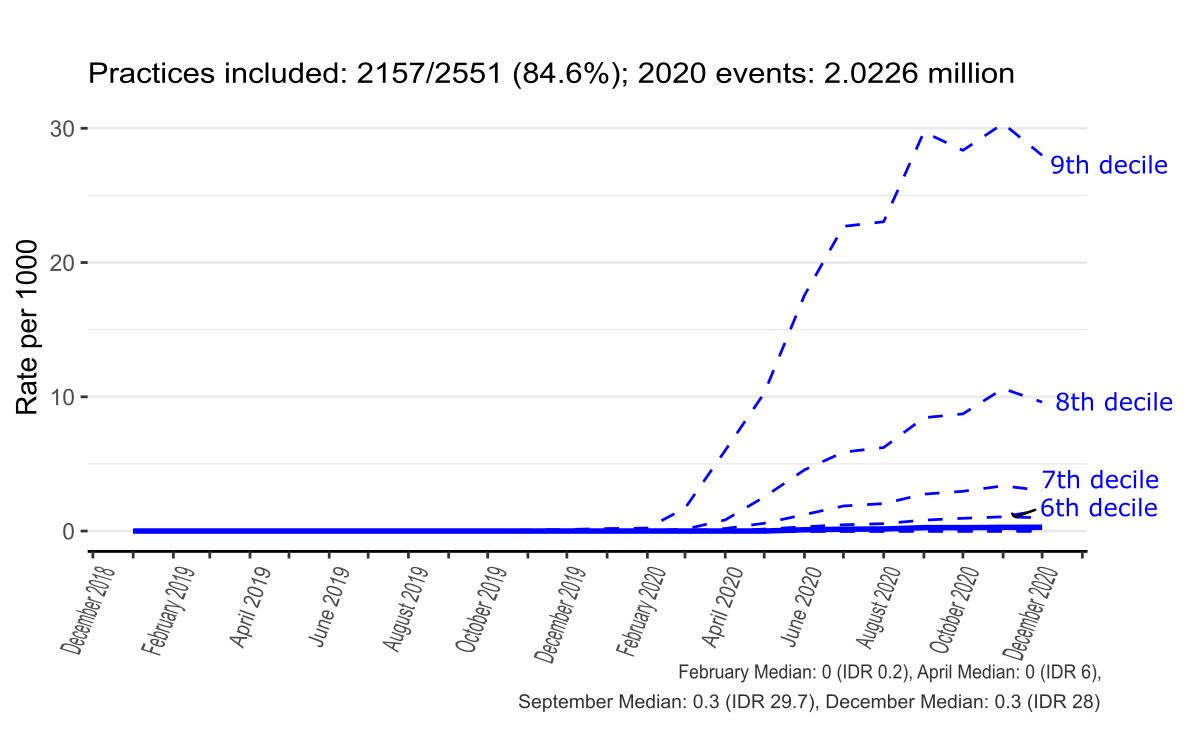
Recording of results from eConsultation via online application (“1068881000000101” - “Y1f3b”) in general practice (January 2019-December 2020). The top 4 deciles can be discerned, with the top practice decile peaking at approximately 30 events per 1000 patients. The lowest 4 deciles of practices have very low rates, which is why they cannot be easily discerned. IDR: interdecile range.

**Figure 6:**
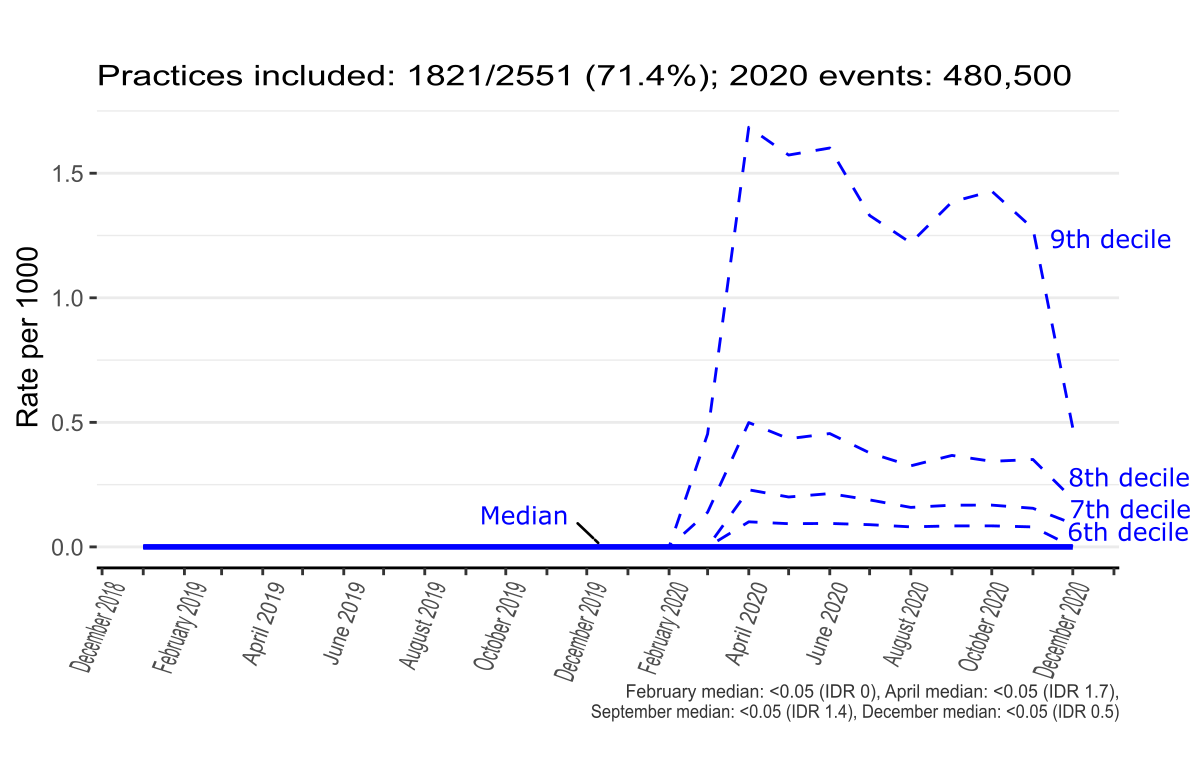
Recording of results from telemedicine consultation with patient (“448337001” “XaXcK”) in general practice (January 2019-December 2020). The top 4 deciles can be discerned, with the top practice decile peaking at >1.5 events per 1000 patients. The lowest 4 deciles of practices have very low rates, which is why they cannot be easily discerned. IDR: interdecile range.

**Figure 7:**
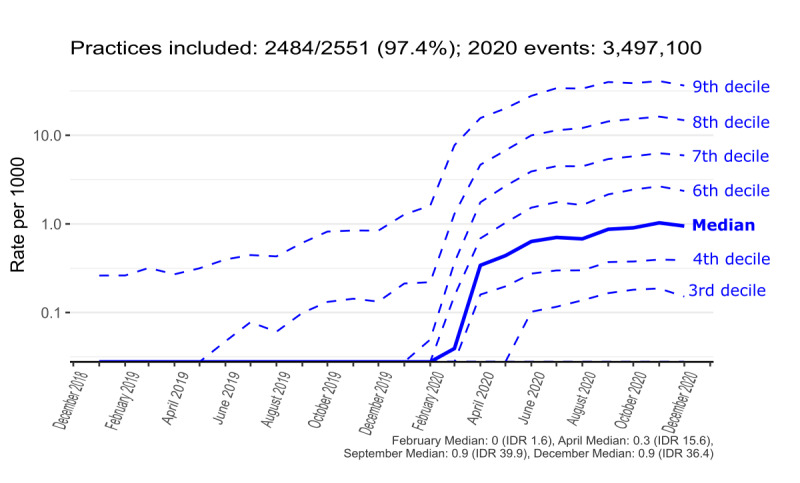
Recording of results from any of the short-listed Systematized Medical Nomenclature for Medicine–Clinical Terminology (SNOMED CT) online consultation codes in general practice (January 2019-December 2020). Logarithmic scale: the top practice decile peaks at >36 events per 1000 patients.

To better convey the coding activity over time and in terms of interpractice variation, the median and decile trends were created for contextual highlighting of GP consultations (Figure 4), *eConsultation* (Figure 5), *telemedicine consultation with patient* (Figure 6), and aggregate of all codes (Figure 7). Deciles illustrate variation across practices for a given metric in a more compact form; for each time point, practices are sorted and ranked from lowest to highest activity, with points that define the top 10%, 20%, ..., 80%, 90% plotted for each month. The 50% decile (ie, the median) is shown as a continuous bold line. Figure 7 shows that there is considerable variation in coding activity levels across practices, with the top deciles of practices gaining much of their activity around the start of the pandemic.

### Demographic Patterns in OC Coding Activity

Broadly, Table 2 shows that the cohort with at least 1 OC-relevant coding instance has a statistically significant higher preponderance of female patients; has a higher relative preponderance of patients aged 18 to 40 years, followed by those aged 40 to 50 years and those aged 50 to 60 years; skews more toward White patients; and skews more toward those who are least deprived. The *P* value (indicating significance) is shown as well as the difference in relative distribution (in percentage points) to understand effect size. Further comparison with the population with GP consultations is given in Tables S1-S7 in Multimedia Appendix 1.

While instances of codes associated with OC systems or remote OCs cannot be compared directly to GP consultation figures or GPAD figures, it is useful to look at relative values across levels of a given sociodemographic characteristic. As such, for further contextualization of the results in Table 2, the distribution of the full study population based on whether they had general practice consultations (as opposed to not) is given in Multimedia Appendix 1 (“Further data: TPP cohort sociodemographic characteristics”). The differential patterns between patients with an OC instance and those with wider general practice interaction, by sociodemographic characteristics, are shown through indicators of rate and coverage. The effect size by individual sociodemographic characteristics is further highlighted in the tables (Multimedia Appendix 1) through standardizing the indicator value for each group of interest by that of the reference group (for ethnicity=White and for deprivation=5 [least deprived]). Patterns are similar to those found when comparing OC activity with the full cohort population in Table 2, albeit in a less pronounced way, both in rates and coverage. Specifically, we still observe a higher relative preponderance of OC-coded activity in patients aged 18 to 40 years and a higher preponderance among those who are least deprived, White, or female. Some other subcohorts are small; therefore, figures and differential patterns require caution in drawing firm conclusions, given the underlying uncertainty.

**Table 2:** Characteristics of the studied cohort, both overall and by (1) patients without a recorded online consultation (OC)–related code instance and (2) patients with such an instance. The *P* value indicates significance for the difference in distributions at the 99.9% CI.

Characteristics		Overall (N=20,651,036)	Had any OC-relevant code instance			*P* value^a^
			No (n=19,563,117)	Yes (n=1,087,919)	Difference in relative breakdown (percentage points)	
**Gender, n (%)**						<.001
	Female	10,260,731 (49.69)	9,599,496 (49.07)	661,235 (60.78)	11.7	
	Male	10,389,976 (50.31)	9,963,322 (50.93)	426,654 (39.22)	−11.7	
	Other or unknown	329 (<0.1)	299 (<0.1)	30 (<0.1)	<0.1	
Age (y), median (IQR)		41 (22-59)	41 (21-59)	43 (27-58)	—^b^	<.001
**Age group (y)**						<.001
	1-18, n (%)	4,298,691 (20.82)	4,151,378 (21.4)	147,313 (13.63)	–7.8	
	18-40, n (%)	5,738,142 (27.79)	5,388,980 (27.78)	349,162 (32.31)	4.5	
	40-50, n (%)	2,842,130 (13.76)	2,665,869 (13.74)	176,261 (16.31)	2.6	
	50-60, n (%)	2,913,528 (14.11)	2,735,067 (14.10)	178,461 (16.52)	2.4	
	60-70, n (%)	2,269,212 (10.84)	2,144,742 (10.90)	124,470 (11.52)	0.6	
	70-80, n (%)	1,673,588 (8.1)	1,598,702 (8.24)	74,886 (6.93)	–1.3	
	>80, n (%)	746,742 (3.62)	716,706 (3.69)	30,036 (2.78)	–0.9	
	Unknown	169,003	161,673	7330	—	
**Ethnicity, n (%)**						<.001
	Asian	1,252,414 (6.06)	1,209,218 (6.18)	43,196 (3.97)	–2.2	
	Black	412,399 (2)	398,242 (2.04)	14,157 (1.3)	–0.7	
	White	12,710,176 (61.55)	11,979,787 (61.24)	730,389 (67.14)	5.9	
	Mixed	249,470 (1.21)	238,762 (1.22)	10,708 (0.98)	–0.2	
	Other	6,026,577 (29.18)	5,737,108 (29.33)	289,469 (26.61)	–2.7	
Living alone, n (%)		5,783,003 (27.91)	5,466,461 (27.84)	316,542 (29.1)	1.3	<.001
**Region**						<.001
	East, n (%)	4,823,404 (23.36)	4,623,066 (23.64)	200,338 (18.42)	–5.2	
	East Midlands, n (%)	3,618,902 (17.53)	3,458,477 (17.68)	160,425 (14.75)	–2.9	
	London, n (%)	1,340,024 (6.49)	1,277,438 (6.53)	62,586 (5.75)	–0.8	
	North East, n (%)	963,807 (4.67)	960,313 (4.91)	3494 (0.32)	–4.6	
	North West, n (%)	1,843,088 (8.93)	1,722,626 (8.81)	120,462 (11.07)	2.3	
	South East, n (%)	1,357,871 (6.58)	1,236,531 (6.32)	121,340 (11.15)	4.8	
	South West, n (%)	2,838,383 (13.75)	2,586,842 (13.23)	251,541 (23.11)	9.9	
	West Midlands, n (%)	861,670 (4.17)	840,558 (4.3)	21,112 (1.94)	–2.4	
	Yorkshire and the Humber, n (%)	2,997,813 (14.52)	2,851,255 (14.58)	146,558 (13.47)	–1.1	
	Unknown	6074	6011	63	—	
**Deprivation quintile**						<.001
	Q1 (most deprived), n (%)	4,157,772 (20.13)	3,989,883 (20.75)	167,889 (15.71)	–5	
	Q2, n (%)	4,032,329 (19.53)	3,822,954 (19.88)	209,375 (29.59)	–0.3	
	Q3, n (%)	4,259,619 (20.63)	4,023,228 (20.92)	236,391 (22.12)	1.2	
	Q4, n (%)	4,052,737 (19.62)	3,817,032 (19.85)	235,705 (22.05)	2.2	
	Q5 (least deprived), n (%)	3,796,821 (18.39)	3,577,294 (18.6)	219,527 (20.54)	1.9	
	Unknown	351,758	332,726	19,032	—	
**Rural or urban, n (%)**						<.001
	Rural	4,113,110 (19.9)	3,896,532 (19.9)	216,578 (19.9)	<0.1	
	Urban	16,209,066 (78.4)	15,355,879 (78.4)	853,187 (78.4)	<0.1	
	Other	328,860 (1.59)	310,706 (1.59)	18,154 (1.67)	<0.1	
**Care home, n (%)**						<.001
	Yes	37,137 (0.18)	34,545 (0.18)	2592 (0.24)	<0.1	
	No	20,613,899 (99.82)	19,528,572 (99.82)	1,085,327 (99.76)	<0.1	

^a^Pearson chi-square test or Wilcoxon rank sum test.

^b^Not applicable.

### Patterns in Clinical History for Patients With eConsultation Coding Activity

The cohort of patients who, during the period from March 2020 to February 2021, had eConsultation activity coded in their records was characterized overall by a lower prevalence (clinical history) of most long-term conditions compared to the remaining population with recorded GP consultation activity during this period (Table 3). Notable exceptions were asthma and depression, where 20.17% (164,868/817,547) and 22.81% (186,523/817,547), respectively, of the eConsultation patients had a clinical history of these compared to 17.21% (1,551,624/9,018,200) and 19.46% (1,754,903/9,018,200), respectively, for other patients with general GP-patient interactions. The comparison against the full population in these practices (rather than just patients therein with GP consultation recorded activity) is given in Table S8 in Multimedia Appendix 1, producing a starker difference for asthma and depression but a more homogeneous profile otherwise (indicating that OC patients resemble more the general population, with respect to clinical history). The tabulation for the *prepandemic* eConsultation activity is given in Table S9 in Multimedia Appendix 1.

From the previous subsection, we have also seen that activity skews to a younger and female-tilted profile, which may be masking some of the clinical history profile characteristics. Table 4 shows that, when considering all clinical history conditions simultaneously, while also factoring in age and gender (first order adjustment), the clinical characteristics that were more prevalent among those with eConsultation activity recorded are those listed in Table 5 (odds ratio >1 and significant). Other clinical history conditions were mainly less prevalent with these adjustments.

**Table 3:** Clinical history characteristics of the cohort with an eConsultation code recorded during the period from March 2020 to February 2021. Comparison against general practitioner consultation activity recorded for the population in those same practices.

Clinical history (before March 2019)	Overall (n=9,835,747), n (%)	Had eConsultation code instance during the period from March 2020 to February 2021 (among those with an eConsultation or general practitioner consultation), n (%)		*P* value^a^
		No (n=9,018,200)	Yes (n=817,547)	
History of hypertension	2,166,059 (22.02)	2,035,381 (22.57)	130,678 (15.98)	<.001
History of asthma	1,716,492 (17.45)	1,551,624 (17.21)	164,868 (20.17)	<.001
History of osteoarthritis	1,519,293 (15.45)	1,426,313 (15.82)	92,980 (11.37)	<.001
History of depression	1,941,426 (19.74)	1,754,903 (19.46)	186,523 (22.81)	<.001
History of diabetes	996,641 (10.13)	938,575 (10.41)	58,066 (7.1)	<.001
History of chronic heart disease	617,213 (6.28)	583,834 (6.47)	33,379 (4.08)	<.001
History of cancer	531,640 (5.41)	497,628 (5.52)	34,012 (4.16)	<.001
History of atrial fibrillation	282,954 (2.88)	268,036 (2,97)	14,918 (1.82)	<.001
History of stroke	197,873 (2.01)	188,013 (2.08)	9860 (1.21)	<.001
History of chronic respiratory disease	404,974 (4.12)	381,487 (4.23)	23,487 (2.87)	<.001
History of peripheral arterial disease	89,347 (0.91)	85,317 (0.95)	4030 (0.49)	<.001
History of heart failure	152,507 (1.55)	144,499 (1.6)	8008 (0.98)	<.001
History of chronic kidney disease	13,393 (0.14)	12,426 (0.14)	967 (0.12)	<.001
History of serious mental illness	114,139 (1.16)	106,557 (1.18)	7582 (0.93)	<.001
Had eConsultation (March 2019-February 2020)	53,521 (0.54)	15,335 (0.17)	38,186 (4.67)	<.001
Had GP consultation (March 2019-February 2020)	8,541,965 (86.85)	7,829,178 (86.82)	712,787 (87.19)	<.001
Had GP consultation (March 2019-February 2020)	9,811,243 (99.75)	9,018,200 (100)	793,043 (97)	<.001

^a^Pearson chi-square test (univariate tests).

**Table 4:** Adjusted odds of having had an online consultation during the period from March 2020 to February 2021, given past clinical history, age, and gender. Odds ratios (ORs) considered against the remaining population in those practices that had any general practitioner consultation or general practitioner–patient interaction recorded during this period.

Characteristics		Adjusted OR (95% CI)	*P* value
Intercept		0.058 (0.057-0.058)	<.001
Male		Reference	—^a^
Female		1.206 (1.200-1.211)	<.001
Gender as other or unknown		1.490 (0.971-2.288)	.07
History of hypertension		1.015 (1.008-1.023)	<.001
History of asthma		1.131 (1.124-1.137)	<.001
History of osteoarthritis		1.048 (1.040-1.057)	<.001
History of depression		1.144 (1.138-1.151)	<.001
History of diabetes		0.858 (0.851-0.866)	<.001
History of chronic heart disease		0.965 (0.953-0.977)	<.001
History of cancer		1.080 (1.068-1.093)	<.001
History of atrial fibrillation		1.119 (1.099-1.139)	<.001
History of stroke		0.914 (0.895-0.933)	<.001
History of chronic respiratory disease		0.928 (0.915-0.941)	<.001
History of peripheral arterial disease		0.897 (0.868-0.926)	<.001
History of heart failure		1.043 (1.018-1.069)	.001
History of chronic kidney disease		0.967 (0.905-1.033)	.31
History of serious mental illness		0.725 (0.708-0.742)	<.001
**Age group (y)**			
	1-18	1.308 (1.295-1.321)	<.001
	18-40	1.940 (1.923-1.957)	<.001
	40-50	1.665 (1.650-1.681)	<.001
	50-60	1.381 (1.369-1.394)	<.001
	60-70	Reference	—
	70-80	0.681 (0.673-0.689)	<.001
	≥80	0.533 (0.524-0.542)	<.001

^a^Not applicable.

**Table 5:** Clinical characteristics that were more prevalent among those with eConsultation activity recorded.

Clinical characteristics	Adjusted odds ratio (95% CI)
Hypertension	1.015 (1.008-1.023)
Asthma	1.131 (1.124-1.137)
Osteoarthritis	1.048 (1.040-1.057)
Depression	1.144 (1.138-1.151)
Cancer	1.080 (1.068-1.093)
Atrial fibrillation	1.119 (1.099-1.139)
Heart failure	1.015 (1.018-1.069)

## Discussion

### Summary

Using OpenSAFELY-TPP and OpenSAFELY-EMIS, we were able to generate data on clinical coding activity relevant to OC systems and remote monitoring across approximately 99% of practices and >53 million patient records. We observed large variation in coding instance rates among practices in England and between the 2 EHR systems. For TPP practices (circa 40% of the practices in England), we explored further the trends and variation in coding activity related to digital forms of interaction. Coding activity increased rapidly during the study period, with a marked increase and acceleration at the start of the pandemic and the first lockdown. Furthermore, we found population subcohorts—both in terms of sociodemographics and clinical history—against which the recorded instance rates were higher.

Above all, this work highlighted that more needs to be done to consolidate and harmonize activity definition (by route and mode) and coding practices associated with the use of OC systems in general practice. Work underway by NHS England (eg, the creation of SNOMED CT codes for OC systems), alongside work to standardize the definitions of demand, capacity, and activity and drive improvements in the user interface of IT systems to support improvements in the consistency of coding and data quality, begin to address this need. As another example, regular open automated reporting could be established via OpenSAFELY, as has been done elsewhere [20], which monitors the coverage and instance rates of relevant codes over time so that central and regional NHS managers or GP practices can review the coding quality over time on its own and through triangulation with local sources or OC system supplier data.

### Findings in the Context and Comparison of Existing Evidence

Of the 18 codes identified, 6 were in active use in TPP practices. The ones used by more practices were, in order, *eConsultation via online application* (2157/2551, 84.56% of practices), *telemedicine consultation with patient* (1822/2551, 71.42%), and *email sent to patient* (1782/2551, 69.85%). In the analysis extension to OpenSAFELY-EMIS, its practices also had registered activity for these codes, but their volumes were quite different, indicating likely differences in the digital systems of different suppliers, coding approaches, or population served. The code *eConsultation via online application* has been explicitly linked to OCs or triages, specifically by supplier eConsult [34] and North of England Commissioning Support in their SystmOne guidance [35]. The code *consultation via multimedia encounter type*, detected in EMIS, has been suggested for use in NHS England total triage guidance [36].

Coding activity related to digital forms of interaction picked up rapidly from the start of the COVID-19 pandemic and the first lockdown (from March 2020 onward). In the second semester of 2020, >9 monthly eConsultation coding events per 1000 registered population were registered compared to <1 per 1000 a year prior. This broad rising trend observed for OC system codes was consistent with the rising trend in weekly OC submissions as captured in the NHS England OC or VC supplier data collection [4], including broadly in terms of the peaks and troughs. Increased adoption and use was expected over the period, given the prepandemic (2019) commitment for all practices to implement and offer OCs and VCs by 2021, with a further legal mandate coming into force in October 2021. The pandemic response likely further accelerated this implementation and also favored increased use, given the substantial shift away from face-to-face appointments due to infection, prevention, and control measures as well as national guidance [36-38]. Although the pandemic was declared over in May 2023 [39], the use of digital forms of GP practice interaction and consultation will continue to be delivered and even likely increase, given the NHS England Delivery Plan for Recovering Access to Primary Care (2023) [6] and the key role played by OC systems in the Modern General Practice Access Model outlined by the plan.

It was noted that NHS England OC or VC and coding event indicators, while similar in trends and patterns, did differ in absolute values. This may relate to a range of reasons, including codes such as eConsultation only being triggered downstream from what is considered a submission in an OC system, certain practices or OC systems not yet using dedicated codes, certain practices or OC systems using codes that are different or broader than those studied here, how an OC submission is defined within the supplier data collection, practices using some codes for clinical activity but not for administrative activity, and certain practices potentially not recording all OC activity in the GP IT clinical system (eg, repeat requests or some practices managing web-based requests and subsequent appointments within their OC system rather than the GP IT clinical system).

Over 2019 and 2020, respectively, 227,429 and 3,323,333 OC system–relevant codes were found in the TPP cohort. When contextualized with wider GP consultation or interaction coding, this corresponded to 1.8 OC system codes for every 1000 GP consultation codes in 2019 and 27.9 OC system codes for every 1000 GP consultation codes in 2020. Although a direct interpretation of this and other publication sources is complicated due to lack of coding standardization, interpretation, and completeness issues, this relative level of OC system use (either route of access or written consultation mode) is broadly in line with observations from other sources. GPAD is the central statistics publication on appointments that have taken place in general practice, showing for instance, record number of >30 million recorded appointments in November 2021. In terms of appointment mode, video, videoconference, or online are all captured under a single category, *video/online*, as per the GPAD specification. This was done to ensure greater consistency between EHR suppliers that submit data for GPAD publication, given the data quality and variability in the ways the submitting suppliers define the mode types, capture them, and report them [40]. In 2019 and 2020, respectively, 5.6 and 4.6 in 1000 appointments had been found to be recorded with respect to mode as either video, videoconference, or online (ie, written consultation), although, as mentioned, publication caveats that are related to how the mode of consultation is recorded and defined, field completion, and the effect of the pandemic warrant caution and could mean that levels are understated and conflated [41]. Much of the partial or total *triage* activity is also not reflected in the collected and published GPAD statistics. Further on appointment mode, in the self-reported GP Patient Survey 2020 and 2021 editions—each mainly covering experiences of the year prior—0.2% and 2.6%, respectively, of those who had booked an appointment said that they received an appointment to speak to someone on the web (eg, video or written) [42].

Interpractice variation was large, reflecting the recent nature of OC system implementation adoption and use: December 2020 saw the median practice have 0.9 recorded codes per 1000 population compared to approximately 36 for the highest decile of practices.

When compared to the full practice population or to patients who had consulted their general practice at all, the cohort of patients who had any recorded coding activity in 2019-2020 tended to skew toward female patients, White patients, and those least deprived. With respect to age, there was a higher relative preponderance of patients aged 18 to 40 years. Although focused on different types of general practice online service access (booking appointments, ordering prescriptions, and viewing records and not an OC system request or consultation), analysis of 2018 and 2019 GP Patient Survey data [43,44] showed analogous evidence of a strong deprivation gradient in the awareness and use of services in favor of those least deprived as well as a reduction in awareness and use for patients aged >75 years. Ethnicity was also associated with variability. A 2019 cross-sectional West Midlands self-administered survey also showed variation in the use and awareness of these 3 services with demographics, namely, lower levels with greater deprivation and with being male (awareness of prescriptions and the awareness and use of online appointment booking) [45]. Patterns by gender and age are also broadly in line with those found in a previous pilot in the South West, using eConsult [13]. The results may also be reflective of the greater implementation challenges, such as the time, capacity, and support required to embed the use of digital tools in practices working in the most challenging circumstances and the highest areas of deprivation, communications, and language to support people to navigate access points and general practice involvement in local commissioning decisions that were made at pace due to the urgency of responding to the pandemic, alongside support with patient factors such as health and digital literacy as well as confidence with, and access to, digital devices and data. Furthermore, there is variation in the design, functionality, and interoperability of OC systems, which may impact the usability and accessibility of different systems. This study does not explore the rates of use across different demographic characteristics between different types of OC systems.

We also found an overall lower prevalence of most long-term conditions in patients who had an eConsultation compared to those with other GP consultation interactions recorded in the same period—in this case, the period from March 2020 to February 2021. In part, this overall lower prevalence may reflect the inherent nature of the intended OC submissions themselves, which are not exclusively focused on the need for a traditional GP consultation but reflect more general population needs such as administrative tasks as well as digitally enabled routine checks and queries.

We found early indications that patients with eConsultation coding activity were more prevalent in cohorts of patients with a clinical history of asthma, depression, or heart conditions when compared to the cohort of other patients with any overall GP consultation activity in the same period. This increased activity may be reflecting not only expected increased general health use in such cohorts but also the prioritized development and adoption (due to the size of the target population or suitability for chronic condition management) of tailored eConsultation solutions and formularies for these cohorts of patients with long-term conditions [46]; for instance, solutions for mental health, self-administered psychometric tests such as the General Anxiety Disorder-7 questionnaire or the Patient Health Questionnaire-9 that can be later reviewed by health professionals, online self-referral routes, and online modes of delivery for talking therapies [47,48] can be supported by, or made interoperable with, an OC system.

More broadly, when considering the aforementioned profiling, it is important to note that the OC system user profile may not be generalizable and may be very dependent on the practice-by-practice model. For instance, the user profile may be influenced by how the OC system has been implemented, to whom and for what conditions the practices have promoted the OC option, the type and design of the OC system, the ease of finding and navigating the OC system, and staff confidence in using digital tools and explaining them to patients. There may also be differences in the user profile in practices with high use of OC systems compared to practices with low use. The authors of the 2016 South West England pilot study did note that the practices involved in the early pilot had fewer patients with long-term health conditions than practices in the rest of England, reflecting potentially greater early-adopter appetite or capability by such practices [13]. Recent studies [49,50] also noted the importance of a complex interplay of multiple determinants of *good access*, including factors such as service responsiveness, flexibility and choice (eg, between digital and nondigital access routes and consultation types), the type and complexity of the problem, continuity, communication, and the wider social context.

### Strengths and Limitations

The key strength of the study relies on the scale and completeness of the underlying record-level EHR data. With OpenSAFELY-TPP, analysis can be run directly on the full data set of raw, single-event clinical events, including tests, treatments, diagnostic events, and other diagnostic and sociodemographic information, covering approximately 40% of the practices in England and equating to >20 million patients, if considering those with a stable practice of registration over the study period. When used along with OpenSAFELY-EMIS, this extends to about 99% of practices. Linkage to secondary care and mortality information is also built in. In comparison with other general practice setting data sources, the Clinical Practice Research Datalink data set holds records on only a sample of patients across 2 databases, while the General Practice Extraction Service data set held by NHS Digital contains fewer data items for each individual patient. NHS England holds a number of record-level commissioning data sets, but these are primarily focused on capturing secondary care activity, specifically across inpatient, outpatient, and emergency settings. NHS England open publications such as GPAD can only give a high-level view of a proportion of activity, with no insight into clinical or sociodemographic factors, quality, or the full workload of general practices. Furthermore, these publications group coarsely all forms of eConsultation as mode of consultation (video or written) and do not provide an easy route to follow GP access types and pathways, although GPAD improvements are being rolled out, including on standardizing appointment categories [51]. Another key strength is the transparency and reproducibility of the analysis undertaken. All code for the platform, data management, and analysis is shared openly on GitHub, allowing for peer review and reusability under open licenses.

In terms of key limitations, as highlighted when describing the codelists in the Methods section, a data-driven approach is taken that relies on SNOMED CT codes and mapping to historical CTV3 hierarchy. Caution must be applied when interpreting the results because not all activity from the use of the OC system is captured in the GP IT clinical system, the codes examined are not specific, and it is unclear whether they are being used to describe either submissions made using an OC system or the consultation mode by written online message or another remote consultation modality. In addition, practices vary in their coding practice, and therefore the use of the codes is unlikely to be consistent. The improvement in coding definition and quality will directly enable potential improvements in insight from OpenSAFELY (and from any other data assets leveraging EHR systems, such as General Practice Extraction Service and Clinical Practice Research Datalink).

### Opportunities for Future Research

The first area to highlight relates to coding quality. By highlighting characteristics and gaps in coding approaches and activity, the insights from this study support work underway with system suppliers to improve coding guidelines and implementation. By regularly monitoring and reviewing the coding activity and reviewing it in context with further data sources and evidence on OC system implementation and use, coding quality could be proactively tracked and improved. This can be done through reduced burden for administrative and clinical staff, given that analysis can be executed in a single framework from re-executable code. As OpenSAFELY encourages curation of thematic and open codelists, the existing codelist can be managed and updated in line with these developments and such that, when relevant, it becomes increasingly aligned with ongoing specification work for GPAD and the forthcoming NHS England OC system supplier data collection being stood up.

Second, forward-looking analysis will be able to leverage insight brought by upcoming SNOMED CT codes that better define and differentiate OC system submissions and written OCs and that differentiate triage in terms of request type (clinical and administrative), the mode of consultation (written OC, video, telephone, and face-to-face), and professional type, allowing for a more journey- or pathway-centric view of digital-first primary care activity. The wider operationalization of OpenSAFELY-EMIS also means that, alongside OpenSAFELY-TPP, studies will be able to draw more comprehensively on data encompassing >99% of the English population [52].

The third area to highlight relates to future opportunities to use OpenSAFELY—alongside a refreshed curated codelist tailored to maximize the tracking of underlying patient activity, namely the code eConsultation—for large-scale cohort or longitudinal studies that help inform continuous improvement and evaluation on service use and patient outcomes. Examples of research questions include the creation of an observatory of inequalities, where a strategic metric is chosen for tracking variation by a key protected characteristic; the characterization of the types of demands and requests submitted by OC system users; and pathway analysis to support impact evaluation protocols (eg, illustratively, the impact of online access and comparisons of modes of consultations on the continuity of care; the rates of accident and emergency department attendance and admissions; secondary care referrals; and general practice appointments use by role and modality, reuse, and prescribing) [9,10,53]. As for the assessment of real-world pilot studies in specific practices (eg, through counterfactual analysis), by design, OpenSAFELY outputs with disclosive practice identifiers cannot be extracted, and querying on practice identifier is not yet straightforward, given pseudonymization. However, there is currently an ongoing cluster randomized controlled trial project where such matching is being carried out in the background by TPP. Subject to ensuring that such functionality would still comply with information governance and data protection—with appropriate processes, controls, and safeguards in place—the ability to define a pilot (intervention) and counterfactual group in the OpenSAFELY study design could in future be extended to external collaborators through simplified platform functionality.

### Conclusions

Insights from this study increase the understanding of the implementation and use of OC systems and written OCs in terms of implementation, trends, and variation. Alongside operational data and evaluation studies, this can support the evidence base around models of OC system implementation and differential patterns of access and uptake. Current gaps in coding practice are also highlighted and can therefore support conversations with practices, OC system suppliers, and EHR suppliers on ensuring consistent and widespread coding practices. Further work that could be leveraged via OpenSAFELY includes key metric monitoring, such as coding quality, coding activity, or variation. Furthermore, the design and implementation of large-scale impact evaluation studies can be considered to understand the types of demands and characteristics of OC system users and how the use of OC systems affects outcomes such as the continuity of care as well as the type and modality of general practice consultation use or unplanned urgent and emergency care.

## Appendix

Multimedia Appendix 1 Supplementary tables and figures.

## Abbreviations

CTV3: Clinical Terms Version 3
DFPC: Digital First Primary Care
EHR: electronic health record
EMIS: Egton Medical Information Systems
GP: general practitioner
GPAD: General Practice Appointments Data
NHS: National Health Service
OC: online consultation
POMI: Patient Online Management Information
SNOMED CT: Systematized Medical Nomenclature for Medicine–Clinical Terminology
TPP: The Phoenix Partnership
VC: video consultation

## Data Availability

The derived data sets generated during this study—that is, aggregate nondisclosive and reviewed outputs—are available in the OS_OC_v001-research github repository [25], under a release-candidate branch. The primary data sets analyzed during this study (detailed pseudonymized patient data) are not publicly available given that they are sensitive and potentially reidentifiable personal information, but can be made available for running new queries and analysis by following the OpenSAFELY framework established in collaboration with National Health Service England. Details of the formal process for external users to request access are available [14]. Further details on information governance can be found in the Ethical Considerations subsection.

All code used for project data management and analysis is shared openly for review and reuse under a Massachusetts Institute of Technology license through a GitHub repository [25]. The developed codelist can be found via the OpenCodelists platform [18].
